# Acid-suppressive medications and risk of colorectal cancer: results from three large prospective cohort studies

**DOI:** 10.1038/s41416-020-0939-y

**Published:** 2020-06-16

**Authors:** Ana Babic, Xuehong Zhang, Vicente Morales-Oyarvide, Chen Yuan, Natalia Khalaf, Hamed Khalili, Paul Lochhead, Andrew T. Chan, Shuji Ogino, Brian M. Wolpin, Kana Wu, Charles S. Fuchs, Edward L. Giovannucci, Meir J. Stampfer, Kimmie Ng

**Affiliations:** 1 Dana-Farber Cancer Institute, Boston, MA USA; 2 Channing Division of Network Medicine, Department of Medicine, Brigham and Women’s Hospital and Harvard Medical School, Boston, MA USA; 3 Division of Gastroenterology, Hepatology, and Endoscopy, Brigham and Women’s Hospital and Harvard Medical School, Boston, MA USA; 4 Clinical and Translational Epidemiology Unit, Department of Medicine, Massachusetts General Hospital, Boston, MA USA; 5 Division of Gastroenterology, Massachusetts General Hospital and Harvard Medical School, Boston, MA USA; 6 Broad Institute of MIT and Harvard, Cambridge, MA USA; 7 Program in MPE Molecular Pathological Epidemiology, Department of Pathology, Brigham and Women’s Hospital and Harvard Medical School, Boston, MA USA; 8 Department of Epidemiology, Harvard T.H. Chan School of Public Health, Boston, MA USA; 9 Department of Nutrition, Harvard T.H. Chan School of Public Health, Boston, MA USA; 10 Yale Cancer Center, New Haven, CT USA; 11 Department of Medicine, Yale School of Medicine, New Haven, CT USA; 12 Smilow Cancer Hospital, New Haven, CT USA

**Keywords:** Epidemiology, Colorectal cancer

## Abstract

**Background:**

Despite several plausible biological mechanisms linking proton pump inhibitors (PPIs) and H2 receptor antagonists (H2RAs) with colorectal tumorigenesis, their association with risk of colorectal cancer (CRC) has not been adequately assessed in prospective epidemiological studies.

**Methods:**

We evaluated the association of acid-suppressive medication use with CRC risk among 175,871 (PPI) and 208,831 (H2RA) participants from three large prospective cohort studies. Medication use was assessed at baseline and updated biennially. The association was evaluated using multivariate Cox proportional hazards regression models.

**Results:**

There was no significant association between baseline PPI use (hazard ratio (HR) = 0.89, 95% confidence interval (CI), 0.71–1.12) or PPI use after a lag of 8–10 years (HR = 1.12, 95% CI, 0.78–1.59) with CRC risk. We observed no significant association between H2RA use after a lag of 8–10 years and CRC risk (HR = 1.02, 95% CI, 0.81–1.28), while risk was lower for participants with baseline H2RA use (HR = 0.76, 95% CI, 0.60–0.95). Duration of PPI use or H2RA use was not associated with CRC risk (*P*-trend = 0.21 and 0.95, respectively).

**Conclusions:**

Among participants from three large prospective cohorts, use of PPI or H2RA was not associated with higher risk of colorectal cancer.

## Background

Acid-suppressive medications, including proton pump inhibitors (PPIs) and histamine type 2 receptor antagonists (H2RAs), are among the most widely used medications in the United States.^1^ These are indicated for the treatment of upper gastrointestinal acid-related disorders, including gastrointestinal reflux disease and peptic ulcer disease,^2^ but lack of adherence to prescription guidelines, as well as over-the-counter availability, have led to their increasing overuse.^3^

Although PPI and H2RA are generally considered safe,^4^ concerns have been raised about long-term use and risk of colorectal cancer. PPI- and H2RA-induced suppression of gastric acid leads to elevated systemic levels of the hormone gastrin,^5–7^ which induces proliferation of colorectal epithelium and colon adenoma progression in animal models.^8–10^ Elevated gastrin levels have been associated with increased colorectal cancer risk in epidemiologic studies.^11^ Second, several recent studies revealed large differences in microbiota composition between PPI users and non-users.^12–14^ Although the relationship between colorectal cancer and the gut microbiota is not yet well understood, several studies strongly implicate specific bacterial species in the development of colorectal tumours.^15,16^ Furthermore, *Fusobacterium nucleatum*, a Gram-negative anaerobic commensal associated with increased colorectal cancer risk,^17,18^ shows in vitro sensitivity to lansoprazole and omeprazole, the two most commonly used PPIs.^19,20^

Despite a potential biological link between acid-suppressive medications and colorectal cancer, this relationship has not been sufficiently investigated in epidemiological studies. Case–control studies suggested increased risk^21,22^ or no association^23–26^ with PPI use, and no association with H2RA use,^21^ but prospective cohort studies are sparse.^27^ Furthermore, previous studies had important limitations, including inadequate adjusting for potential confounders, such as history of colonoscopy and BMI, small number of cases and a relatively short duration of follow-up. Most importantly, since colorectal cancer has a long induction period of at least 10 years,^28^ it is crucial to carefully examine timing of exposure in relation to cancer diagnosis. We therefore conducted a prospective analysis to evaluate the association between use of acid-suppressive medications and colorectal cancer risk within three large cohorts (Nurses’ Health Study [NHS], Nurses’ Health Study II [NHSII] and Health Professionals Follow-Up Study [HPFS]) with updated information on PPI and H2RA use, with up to 26 years of follow-up.

## Methods

### Study population

The NHS, NHSII and HPFS are ongoing US-based prospective cohort studies. The NHS was established in 1976 among 121,700 US female registered nurses aged 30–55 years,^29,30^ and NHSII was established in 1989 among 116,429 US female nurses aged 25–42 years.^31^ HPFS was established in 1986 among 51,529 US male health professionals aged 40–75 years at baseline.^32^ In all three cohorts, participants completed an initial questionnaire about their medical history, lifestyle factors and health behaviours. Participants have been mailed follow-up questionnaires every 2 years to provide updated information on those factors, as well as disease outcomes. Participants were also asked to complete semi-quantitative validated food frequency questionnaires (FFQs) approximately every 4 years. Follow-up is >90% for all three cohorts. The study protocol was approved by the institutional review boards of the Brigham and Women’s Hospital and Harvard T.H. Chan School of Public Health, and those of participating registries as required.

The eligible population for the analysis of PPI use consisted of participants who answered the questionnaire in which PPI use was first assessed (NHS, 2000; NHSII, 2001; HPFS, 2004). We excluded participants with previous history of cancer, except non-melanoma skin cancer and history of ulcerative colitis or Crohn’s disease. After those exclusions, a total of 175,871 participants were included in the analysis. Among eligible participants who answered the first questionnaire where H2RA use was assessed (NHS, 1994; NHSII, 1995; HPFS, 1988), we applied the same exclusions, leaving a total of 208,831 participants.

### Ascertainment of PPI and H2RA use

Information about PPI use and H2RA use was obtained from the baseline questionnaire, and updated in each subsequent biennial cycle. At baseline, participants were asked about regular use (yes/no) of Prilosec or Prevacid in NHS and NHSII, and Prilosec, Nexium, Prevacid (lansoprazole), Protonix or Aciphex in HPFS in the past 2 years. Similarly, they were asked about regular use of ranitidine (Zantac) or cimetidine (Tagamet) in NHS, cimetidine (Tagamet) or other H2 blockers (e.g. Zantac, Pepcid, Axid) in NHSII, and cimetidine or ranitidine (e.g. Tagamet, Zantac) in HPFS in the past 2 years. If a participant’s information on medication use was missing for a follow-up cycle, then that participant did not contribute person-time to the analysis for that cycle.

### Ascertainment of covariates

Information about demographic and lifestyle factors known to be associated with colorectal cancer was obtained from the baseline and follow-up questionnaires. These included age, height, weight, physical activity, family history of colorectal cancer, regular use of aspirin and non-aspirin nonsteroidal anti-inflammatory drugs (NSAIDs), menopausal status, menopausal hormone therapy (MHT) use, multivitamin use and history of lower endoscopy (including indication for the procedure). All the covariates were updated biennially, except for family history of colorectal cancer, which was updated approximately every 4 years. Once a participant reported an endoscopy, positive history of endoscopy was carried forward in all the subsequent cycles. Information on dietary factors, including intake of fibre, vitamin D, calcium, folate, red meat, processed meat, total fat intake and total caloric intake, was extracted from FFQs every 4 years. We used energy-adjusted values and cumulative updated intake, defined as the average intake from all available FFQs up to the most recent biennial follow-up questionnaire. Participants were asked about a history of gastric or duodenal ulcer in NHS only at two time points (2000, 2008); in the other two cohorts, this information was updated every 2 years after the baseline (1991 in NHSII, 1986 in HPFS).

### Documentation of colorectal cancer cases and deaths

Information about disease outcomes, including colorectal cancer, was obtained in biennial questionnaires. For each participant reporting a colorectal cancer diagnosis, we asked for permission to obtain medical records and pathology reports. Deaths of participants were identified through family members, National Death Index or the US postal service. If the review of death certificate revealed colorectal cancer as the cause of death, we sought permission from next of kin to obtain and review medical records. A study physician blinded to exposure status reviewed medical records, and extracted information on cancer stage, histopathologic features and tumour location.

### Statistical analysis

Due to the similar questionnaire design across cohorts, we combined data from all three cohorts into a single dataset. Person-time was calculated as time from the return of baseline questionnaires until colorectal cancer diagnosis, diagnosis of any cancer, except non-melanoma skin cancer, death or the end of follow-up (NHS: June 2014; NHSII: June 2015; HPFS: January 2014), whichever came first.

To consider different intervals between exposures and outcome, we used several approaches to modelling exposures, including current, baseline, persistent, cumulative and lagged use of PPI and H2RA. Current use was defined as regular medication use reported in the most recent questionnaire; this variable was updated in each biennial follow-up cycle. Baseline use was assessed on the first questionnaire that asked about PPI or H2RA use, and subsequently carried forward. Persistent use was defined as use reported in the baseline questionnaire plus one or two subsequent follow-up questionnaire cycles. To evaluate the cumulative effect of drug use on cancer risk, we assessed the number of cycles with reported drug use (no use, use in 1–2 cycles, use in ≥3 cycles), regardless of whether these cycles were consecutive or not. Lastly, since the association between several established risk factors and colorectal cancer is observed only after a latency of 10 years or longer,^33^ we examined medication use lagged for varying periods of time up to 10 years. For example, in NHS, PPI use was first assessed in 2000. For a latency of 2–4 years, we used PPI use in 2000 for the follow-up period from 2002 to 2004, PPI use in 2002 for the follow-up period from 2004 to 2006 and so forth. For a latency of 4–6 years, we used PPI use in 2000 for the follow-up period from 2004 to 2006, PPI use in 2002 for the follow-up period from 2006 to 2008 and so on.

We used Cox proportional hazards regression models stratified by age (months) and 2-year questionnaire cycle to estimate the hazard ratios (HRs) and 95% confidence intervals (CIs) of cancer risk for users of acid-suppressive medications, compared to non-users. In the main multivariate model, we adjusted for study cohort (NHS, NHSII, HPFS) and established colorectal cancer risk factors, including BMI (<25 kg/m^2^, 25–27.5 kg/m^2^, 27.5–30 kg/m^2^, ≥30 kg/m^2^), physical activity (<3 metabolic equivalent [MET]-h/week, 3–27 MET-h/week, ≥27 MET-h/week), family history of colorectal cancer (no, yes), daily caloric intake (quintiles), alcohol intake (<5 g/day, 5–15 g/day, ≥15 g/day), pack-years of smoking (0, 1–10, ≥10 pack-years), history of lower endoscopy (never, ever), dietary and supplemental vitamin D intake (quintiles), dietary and supplemental calcium intake (quintiles), regular aspirin use (no, yes), dietary and supplemental folate intake (quintiles), MHT use (premenopausal, postmenopausal never MHT user, postmenopausal past MHT user, postmenopausal current MHT user) (NHS and NHSII only) and red meat intake (quintiles). We additionally examined confounding by waist circumference (continuous), waist-to-hip ratio (continuous), regular NSAID use (no, yes) and dietary intake of processed meat (quintiles). To evaluate the heterogeneity of the association by cohort, we pooled the cohort-specific estimates, and calculated the *Q*-statistic using random-effects meta-analysis.^34^ We performed stratified analyses by gender, history of lower endoscopy (never, ever), BMI (<25 kg/m^2^, 25–30 kg/m^2^, ≥30 kg/m^2^) and history of smoking (never, ever) to identify effect modification. We included a cross-product between PPI or H2RA use and stratifying variable in the multivariate model, and tested the significance of interaction using the Wald test. All *P* values were two-sided, using a 0.05 significance level. All the analyses were performed using the SAS software (SAS Institute, Inc., Version 9.4).

## Results

We documented 1255 colorectal cancer cases during 2.1 million years of follow-up in the PPI use analysis, and 2413 cases during 3.8 million years of follow-up in the H2RA use analysis.

Compared to their respective non-users, both PPI and H2RA users had on average a higher BMI, lower levels of physical activity and higher total calcium intake. Users were more likely to have a history of lower endoscopy, in particular due to gastrointestinal symptoms, and use aspirin, NSAID and multivitamins (Tables 1 and 2). Among women, PPI and H2RA users were more likely to be postmenopausal and MHT users. There were fewer never smokers among both PPI and H2RA users.

**Table 1:** Baseline characteristics of participants by use of proton pump inhibitors.

Characteristic^a^	Nurses’ Health Study (2000) Nurses’ Health Study II (2001)		Health Professionals Follow-Up Study (2004)	
	Non-users (*n* = 141,809)	Users (*n* = 9368)	Non-users (*n* = 20,845)	Users (*n* = 3837)
Age (years)^b^	56 (11)	57 (11)	69 (9)	70 (9)
Body mass index (kg/m^2^)	25 (5)	27 (6)	26 (3)	26 (3)
Activity (MET-h/week)	19 (19)	16 (16)	35 (28)	32 (26)
Total folate intake (µg/day)	462 (212)	458 (205)	609 (231)	623 (233)
Total vitamin D (IU/day)	372 (201)	377 (198)	446 (228)	461 (234)
Total calcium intake (mg/day)	1058 (388)	1081 (403)	969 (339)	992 (355)
Total fat (g/day)	60 (10)	60 (10)	68 (12)	69 (12)
Total fibre (g/day)	19 (5)	18 (5)	23 (6)	22 (6)
Alcohol (g/day)	5 (7)	4 (7)	11 (13)	11 (13)
Total energy intake (kcal/day)	1767 (465)	1778 (473)	2000 (531)	1989 (534)
Red meat (servings/week)	4 (2)	4 (2)	4 (3)	4 (3)
Processed meat (servings/week)	2 (2)	2 (2)	2 (2)	2 (2)
History of sigmoidoscopy/colonoscopy	39	55	73	84
Family history of colorectal cancer	13	14	13	14
H2RA use	6	11	5	14
Regular aspirin use (≥2 tablets/week)	27	35	49	53
Regular NSAID use (≥2 tablets/week)	33	44	29	39
Multivitamin use	62	63	68	71
White	97	98	96	97
Menopausal hormone therapy use	40	48	N/A	N/A
Postmenopausal status	55	59	N/A	N/A
History of smoking				
Never	56	52	53	49
Past	35	39	44	48
Current	9	9	4	3

*H2RA* H2 receptor antagonist, *MET* metabolic equivalent, *NSAID* nonsteroidal anti-inflammatory drugs.

^a^Age-adjusted means (standard deviation) for continuous variables, and percentages for categorical variables.

^b^Values not age adjusted.

**Table 2:** Baseline characteristics of participants by use of H2 receptor antagonists.

Characteristic^a^	Nurses’ Health Study (1994) Nurses’ Health Study II (1995)		Health Professionals Follow-up Study (1988)	
	Non-users (*n* = 162,473)	Users (*n* = 9001)	Non-users (*n* = 36,433)	Users (*n* = 1008)
Age (years^b^)	50 (12)	52 (11)	56 (10)	59 (10)
Body mass index (kg/m^2^)	25 (5)	27 (6)	26 (3)	26 (3)
Activity (MET-h/week)	20 (24)	18 (22)	25 (27)	20 (22)
Total folate intake, (µg/day)	441 (211)	438 (210)	480 (273)	461 (261)
Total vitamin D (IU/day)	364 (211)	374 (220)	405 (307)	406 (279)
Total calcium intake (mg/day)	993 (375)	1023 (398)	902 (418)	909 (409)
Total fat (g/day)	60 (10)	61 (10)	71 (14)	72 (14)
Total fibre (g/day)	18 (5)	18 (5)	21 (7)	20 (6)
Alcohol (g/day)	4 (7)	4 (7)	11 (15)	12 (15)
Total energy intake (kcal/day)	1760 (473)	1787 (492)	1995 (616)	2040 (621)
Red meat (servings/week)	4 (2)	4 (3)	4 (3)	5 (4)
Processed meat (servings/week)	2 (2)	2 (2)	3 (3)	3 (3)
History of sigmoidoscopy/colonoscopy	25	37	24	32
Family history of colorectal cancer	10	11	9	9
Regular aspirin use (≥2 tablets/week)	23	31	41	44
Regular NSAID use (≥2 tablets/week)	26	43	10	21
Multivitamin use	44	45	41	44
White	97	97	96	97
Menopausal hormone therapy use	29	37	N/A	N/A
Postmenopausal status	43	47	N/A	N/A
History of smoking				
Never	56	51	49	40
Past	32	35	42	49
Current	12	14	8	12

*MET* metabolic equivalent, *NSAID* nonsteroidal anti-inflammatory drugs.

^a^Age-adjusted means (standard deviation) for continuous variables, and percentages for categorical variables.

^b^Values not age adjusted.

In multivariate adjusted models, there was no significant association between baseline use of PPI and risk of colorectal cancer (HR = 0.89, 95% CI, 0.71–1.12), while current PPI use was associated with decreased risk (HR = 0.82, 95% CI, 0.68–0.98) (Table 3). Persistent PPI use defined as use in the baseline, and one (HR = 0.83, 95% CI, 0.58–1.18) or two subsequent cycles (HR = 0.84, 95% CI, 0.56–1.24) was not significantly associated with risk (Table 3). We next evaluated cumulative PPI use, and observed no significant association between number of cycles with reported PPI use and risk of colorectal cancer (*P*-trend = 0.21) (Table 4). To examine different latency periods between PPI use and colorectal cancer diagnosis, we lagged PPI use for up to 10 years, and observed no significant association (8–10 years lag: HR = 1.12, 95% CI, 0.78–1.59) (Table 5).

**Table 3:** Association between use of acid-suppressive medications and risk of colorectal cancer.

	Nurses’ Health Study Nurses’ Health Study II		Health Professionals Follow-Up Study		Combined	
	Non-users	Users	Non-users	Users	Non-users	Users
Proton pump inhibitors use						
Baseline use						
No. of cases	998	53	174	30	1172	83
Person-years	1,785,384	115,921	172,283	31,207	1,957,667	147,128
Age-adjusted HR (95% CI)	Ref.	0.78 (0.59–1.02)	Ref.	0.92 (0.62–1.36)	Ref.	0.84 (0.67–1.04)
Multivariate HR (95% CI)^a^	Ref.	0.86 (0.65–1.14)	Ref.	1.02 (0.68–1.52)	Ref.	0.89 (0.71–1.12)
Use in two consecutive baseline cycles						
No. of cases	1027	20	187	13	1214	33
Person-years	1,831,700	53,137	182,512	17,897	2,014,212	71,034
Age-adjusted HR (95% CI)	Ref	0.77 (0.49–1.20)	Ref.	0.71 (0.40–1.25)	Ref.	0.76 (0.55–1.10)
Multivariate HR (95% CI)^a^	Ref.	0.87 (0.55–1.35)	Ref.	0.81 (0.45–1.44)	Ref.	0.83 (0.58–1.18)
Use in three consecutive baseline cycles						
No. of cases	1030	16	190	10	1220	26
Person-years	1,839,152	43,172	186,371	13,010	2,025,523	56,182
Age-adjusted HR (95% CI)	Ref.	0.75 (0.46–1.23)	Ref.	0.74 (0.39–1.41)	Ref.	0.77 (0.52–1.14)
Multivariate HR (95% CI)^a^	Ref.	0.85 (0.52–1.40)	Ref.	0.84 (0.44–1.62)	Ref.	0.84 (0.56–1.24)
Current use						
No. of cases	873	111	159	26	1032	137
Person-years	1,472,450	245,294	152,585	28,702	1,625,035	273,996
Age-adjusted HR (95% CI)	Ref.	0.72 (0.59–0.88)	Ref.	0.83 (0.54–1.27)	Ref.	0.73 (0.61–0.88)
Multivariate HR (95% CI)^a^	Ref.	0.80 (0.65–0.98)	Ref.	0.91 (0.59–1.41)	Ref.	0.82 (0.68–0.98)
H2 receptor antagonists use						
Baseline use						
No. of cases	1486	56	837	22	2323	78
Person-years	2,881,874	153,014	705,765	17,711	3,587,639	170,724
Age-adjusted HR (95% CI)	Ref.	0.66 (0.51–0.87)	Ref.	0.96 (0.63–1.48)	Ref.	0.71 (0.56–0.88)
Multivariate HR (95% CI)^a^	Ref.	0.70 (0.53–0.92)	Ref.	1.01 (0.65–1.55)	Ref.	0.76 (0.60–0.95)
Use in two consecutive baseline cycles						
No. of cases	1495	42	771	11	2266	53
Person-years	2,928,762	91,273	647,808	7706	3,576,570	98,979
Age-adjusted HR (95% CI)	Ref.	0.80 (0.59–1.09)	Ref.	1.12 (0.61–2.05)	Ref.	0.81 (0.62–1.07)
Multivariate HR (95% CI)^a^	Ref.	0.85 (0.62–1.16)	Ref.	1.24 (0.67–2.27)	Ref.	0.90 (0.68–1.18)
Use in three consecutive baseline cycles^b^						
No. of cases	1213	20	699	7	1912	27
Person-years	1,285,353	29,968	607,977	5463	1,893,330	35,430
Age-adjusted HR (95% CI)	Ref.	0.69 (0.44–1.07)	Ref.	0.96 (0.45–2.05)	Ref.	0.72 (0.49–1.05)
Multivariate HR (95% CI)^a^	Ref.	0.73 (0.47–1.14)	Ref.	1.08 (0.51–2.31)	Ref.	0.79 (0.54–1.15)
Current use						
No. of cases	1294	102	699	45	1993	147
Person-years	2,333,665	173,958	603,823	31,002	2,937,488	204,960
Age-adjusted HR (95% CI)	Ref.	0.94 (0.77–1.15)	Ref	1.15 (0.85–1.56)	Ref.	0.98 (0.83–1.16)
Multivariate HR (95% CI)^a^	Ref.	1.00 (0.81–1.22)	Ref	1.23 (0.90–1.67)	Ref.	1.06 (0.89–1.26)

*BMI* body mass index, *CI* confidence interval, *HR* hazards ratio, *MHT* menopausal hormone therapy, *NHS* Nurses’ Health Study, *NHSII* Nurses’ Health Study II.

^a^Adjusted for age, BMI ( < 25 kg/m^2^, 25–27.5 kg/m^2^, 27.5–30 kg/m^2^, ≥30 kg/m^2^), physical activity (<3 MET-h/week, 3–27 MET-h/week, ≥27 MET-h/week), family history of colorectal cancer (no, yes), alcohol intake (<5 g/day, 5–15 g/day, ≥15 g/day), pack-years of smoking (0, 1–10, ≥10 pack-years), history of lower endoscopy (never/ever), caloric intake (quintiles), vitamin D (quintiles), calcium intake (quintiles), regular aspirin use (no, yes), folate intake (quintiles), MHT use (premenopausal, postmenopausal never user, postmenopausal past user, postmenopausal current user) (NHS and NHSII only) and red meat as main dish (quintiles).

^b^Evaluated in NHS and HPFS only.

**Table 4:** Duration of acid-suppressive medications use and risk of colorectal cancer.

	No use	Used in 1–2 cycles	Used in ≥3 cycles	*P*-trend
PPI use				
No. of cases	952	159	58	
Person-years	1,493,933	288,870	116,229	
Age-adjusted HR (95% CI)	Ref.	0.80 (0.68–0.95)	0.79 (0.60–1.04)	0.01
Multivariate HR (95% CI)^a^	Ref.	0.89 (0.75–1.06)	0.92 (0.69–1.21)	0.21
H2RA use				
No. of cases	1821	225	94	
Person-years	2,652,702	378,358	111,387	
Age-adjusted HR (95% CI)	Ref.	0.84 (0.73–0.97)	1.02 (0.82–1.25)	0.33
Multivariate HR (95% CI)^a^	Ref.	0.91 (0.79–1.05)	1.09 (0.88–1.35)	0.95

*BMI* body mass index, *CI* confidence interval, *HR* hazards ratio, *MHT* menopausal hormone therapy, *NHS* Nurses’ Health Study, *NHSII* Nurses’ Health Study II.

^a^Adjusted for age, BMI ( < 25 kg/m^2^, 25–27.5 kg/m^2^, 27.5–30 kg/m^2^, ≥30 kg/m^2^) physical activity (<3 MET-h/week, 3–27 MET-h/week, ≥27 MET-h/week), family history of colorectal cancer (no, yes), alcohol intake (<5 g/day, 5–15 g/day, ≥15 g/day), pack-years of smoking (0, 1–10, ≥10 pack-years), history of lower endoscopy (never/ever), caloric intake (quintiles), vitamin D (quintiles), calcium intake (quintiles), regular aspirin use (no, yes), folate intake (quintiles), MHT use (premenopausal, postmenopausal never user, postmenopausal past user, postmenopausal current user) (NHS and NHSII only) and red meat as main dish (quintiles).

**Table 5:** Association between colorectal cancer and lagged use of acid-suppressive medications.

	2–4 years lag		4–6 years lag		6–8 years lag		8–10 years lag	
	Non-users	Users	Non-users	Users	Non-users	Users	Non-users	Users
PPI use								
No. of cases	831	108	662	84	496	47	331	36
Person-years	1,373,645	219,447	1,118,996	163,824	877,318	115,076	631,274	69,835
Age-adjusted HR (95% CI)	Ref.	0.76 (0.62 to 0.94)	Ref.	0.81 (0.65 to 1.03)	Ref.	0.72 (0.53 to 0.97)	Ref.	1.01 (0.71 to 1.44)
Multivariate HR (95% CI)^a^	Ref.	0.85 (0.69 to 1.05)	Ref.	0.90 (0.71 to 1.14)	Ref.	0.78 (0.58 to 1.07)	Ref.	1.12 (0.78 to 1.59)
H2RA use								
No. of cases	1857	126	1650	99	1430	88	1180	78
Person-years	2,615,912	179,320	2,274,028	153,533	1,954,486	131,335	1,639,395	109,876
Age-adjusted HR (95% CI)	Ref.	0.92 (0.76–1.10)	Ref.	0.82 (0.67–1.01)	Ref.	0.87 (0.70–1.08)	Ref.	0.96 (0.76–1.21)
Multivariate HR (95% CI)^a^	Ref.	0.99 (0.83–1.19)	Ref.	0.89 (0.72–1.09)	Ref.	0.95 (0.76–1.18)	Ref.	1.02 (0.81–1.28)

*BMI* body mass index, *CI* confidence interval, *HR* hazards ratio, *MHT* menopausal hormone therapy, *NHS* Nurses’ Health Study, *NHSII* Nurses’ Health Study II.

^a^Adjusted for age, BMI ( < 25 kg/m^2^, 25–27.5 kg/m^2^, 27.5–30 kg/m^2^, ≥30 kg/m^2^) physical activity (<3 MET-h/week, 3–27 MET-h/week, ≥27 MET-h/week), family history of colorectal cancer (no, yes), alcohol intake (<5 g/day, 5–15 g/day, ≥15 g/day), pack-years of smoking (0, 1–10, ≥10 pack-years), history of lower endoscopy (never/ever), caloric intake (quintiles), vitamin D (quintiles), calcium intake (quintiles), regular aspirin use (no, yes), folate intake (quintiles), MHT use (premenopausal, postmenopausal never user, postmenopausal past user, postmenopausal current user) (NHS and NHSII only) and red meat as main dish (quintiles).

Use of H2RA at baseline was associated with decreased risk of colorectal cancer (HR = 0.76, 95% CI, 0.60–0.95), while there was no significant association between current or persistent H2RA use (Table 3). Duration of H2RA use was also not significantly associated with risk (*P*-trend = 0.95) (Table 4). We next examined several latency periods up to 10 years, and observed no significant association (8–10 years lag: HR = 1.02, 95% CI, 0.81–1.28) (Table 5).

The associations between PPI use and H2RA use and colorectal cancer risk each remained materially unchanged after adjusting for additional potential confounders, including waist circumference, waist-to-hip ratio, NSAID use, intake of processed meat and history of gastric or duodenal ulcer (data not shown). Associations between both medications and risk of colorectal cancer were similar by primary tumour location (Supplementary Table 1) and by study cohort (*P*-heterogeneity ≥0.13). Furthermore, the associations for both medications and colorectal cancer risk were similar in analyses stratified by sex, history of lower endoscopy, BMI and smoking status (all *P*-interaction ≥0.24) (Supplementary Table 2). Since PPI users were also more likely to use H2RA (Table 1), we adjusted simultaneously for use of both medications to examine their independent association with colorectal cancer. Neither baseline use of PPI (HR = 0.95, 95% CI, 0.75–1.21) nor H2RA (HR = 0.82, 95% CI, 0.59–1.12) was associated with risk of colorectal cancer in these models.

## Discussion

In this pooled analysis of three large prospective US cohorts, we found no evidence of overall significant association between use of PPI or H2RA medications and risk of colorectal cancer.

Use of both PPI and H2RA was examined using several approaches, with varying latency periods between medication use and colorectal cancer diagnosis. In the analysis of PPI use, we observed no significant association with baseline, persistent, cumulative or up to 10-year lagged medication use and colorectal cancer risk. We observed decreased colorectal cancer risk in current users of PPI; however, use in the past 2 years before diagnosis is not biologically likely to play a role in disease development, and is more likely to be affected by subclinical disease. There was no significant change in colorectal cancer risk in current H2RA users. We did observe a 23% decrease in colorectal cancer risk that was statistically significant among baseline H2RA users. However, we observed no association with persistent use or longer duration use, arguing against the inverse association between H2RA and colorectal cancer being causal. Overall, these observations do not support the causal role of PPI or H2RA in colorectal cancer aetiology.

Several case–control studies have examined PPI use in relation to colorectal cancer risk, and reported either no association^23–26^ or moderately increased risk among PPI users.^21,22^ One case–control study reported no association between H2RA use and risk of colorectal cancer.^21^ While most of those studies relied on information about medication use extracted from health insurance records or medical charts; therefore, eliminating recall bias, over-the-counter use of these medications was not considered, which could lead to exposure misclassification. Furthermore, several studies failed to account for important confounders such as BMI or history of colonoscopy.^21–25^ In our study, users of PPI and H2RA had a higher BMI and were more likely to have a history of colonoscopy, which have been associated with increased or decreased risk of CRC, respectively.^35,36^ Most importantly, in previous studies timing of medication use in relation to colorectal cancer diagnosis was not carefully examined. Colorectal cancer has a long latency period of 10 or more years, and a long latency period was required to observe the protective effect of aspirin in prospective cohort studies.^37^ We considered several exposure windows, and observed no association with risk of colorectal cancer with medication use lagged up to 10 years. Furthermore, overall duration of follow-up in our study was up to 26 years, compared with up to 15 years in previous studies,^26^ additionally increasing our ability to detect any potential association.

To our knowledge, this is the first prospective cohort study to evaluate H2RA use in relation to risk of colorectal cancer. One previous prospective cohort study also found no overall increase in colorectal cancer risk in PPI users;^27^ however, this study had unclear time interval between exposure assessment and beginning of follow-up, lacked information on exposure and covariate updating, and did not consider some important confounders, such as history of colonoscopy. We were able to adjust for multiple confounders and examine multiple classifications of updated medication use, and we found no evidence of significant association between PPI use and risk of colorectal cancer.

Both PPI and H2RA lead to increased circulating levels of gastrin,^5–7^ which has been associated with increased risk of colorectal cancer in prospective studies.^11^ In animal models of colorectal cancer, gastrin leads to increased proliferation of colon mucosa and adenoma progression.^8–10^ It is possible that the rise in gastrin levels due to PPI and H2RA use is not sufficient to promote development of colorectal tumours. Higher doses of medication over long periods of time may be necessary to exert an effect on tumour progression. While information on medication dosing was not available in our study, we observed no association with longer duration of use of acid-reducing medications with colorectal cancer. We also observed no increase in risk among patients with consistent PPI and H2RA use.

Furthermore, use of acid-suppressive medications leads to inhibition of gastric acid secretion and increased gastric pH, altering both the gastric microbiome and gut microbiome.^38^ Recent studies have revealed significant differences in the composition of the gut microbiome between PPI users and non-users,^12–14^ and growing evidence suggests a role of an altered microbiome in colorectal cancer development.^15,16^ However, it is not clear whether microbiome changes related to PPI use are overlapping with those associated with colorectal cancer. Our results do not suggest an adverse effect of microbiome changes caused by PPI use on colorectal cancer development.

A strength of this study is the prospective design that avoids the recall bias that often occurs in case–control studies, the large number of colorectal cancer cases and long follow-up in three independent cohorts of men and women. We collected information on the use of two acid-suppressive medications with different mechanisms of action; this use was updated every 2 years. Due to extensive information about lifestyle and dietary factors, we were able to adjust for important variables associated with both medication use and colorectal cancer, thereby minimising potential confounding. We were also able to evaluate confounding by indication, by accounting in our analyses for peptic or gastric ulcers, common reasons for use of acid-suppressive medications.

This study has several limitations. Since the questionnaire asked about “regular use” of the acid-suppressive medications in the previous 2-year cycle, without specifying the dose or frequency, we were not able to investigate a more precise dose–response relationship between acid-suppressive medication use and colorectal cancer. We had limited power to examine in more detail the associations with different tumour locations. Furthermore, evaluation of an even longer latency period might be necessary to further confirm the observed lack of association. Most of the participants in our cohort study were white, and our observations may therefore not be generalisable to other races and/or ethnicities.

In conclusion, in this prospective analysis among participants of three large cohorts, we observed no association between use of acid-suppressive medications and risk of colorectal cancer. Given the highly prevalent use of acid-suppressive medications in the US population, the lack of an association with increased colorectal cancer risk is reassuring.

## Supplementary information

Supplementary Files

## Data Availability

Information on obtaining access to NHS, NHSII and HPFS data is available at https://www.nurseshealthstudy.org/researchers (email: nhsaccess@channing.harvard.edu) and https://sites.sph.harvard.edu/hpfs/for-collaborators.
