# Supplementary material for: Acid-suppressive medications and risk of colorectal cancer: results from three large prospective cohort studies
Source: Br J Cancer. 2020 Jun 16;123(5):844–51. doi: 10.1038/s41416-020-0939-y (PMC7462971; doi:10.1038/s41416-020-0939-y)
Supplement: Supplementary file 1 — Supplementary Files [file 41416_2020_939_MOESM1_ESM.docx]

**Supplementary Table 1**. Baseline use of acid suppressive medications and risk of colorectal cancer by tumor location

|  | **Colon** | | **Rectum** | |
| --- | --- | --- | --- | --- |
|  | Non-users | Users | Non-users | Users |
| **PPI use** |  |  |  |  |
| N cases | 923 | 69 | 249 | 14 |
| Pearson-years | 1,957,880 | 147,148 | 1,958,471 | 147,195 |
| Age-adjusted HR (95% CI) | Ref. | 0.87 (0.68 to 1.11) | Ref. | 0.71 (0.41 to 1.22) |
| Multivariate HR (95% CI)* | Ref. | 0.91 (0.71 to 1.16) | Ref. | 0.82 (0.47 to 1.41) |
| **H2RA use** |  |  |  |  |
| N cases | 1,805 | 62 | 518 | 16 |
| Person-years | 3,588,090 | 170,739 | 3,589,252 | 170,776 |
| Age-adjusted HR (95% CI) | Ref. | 0.72 (0.55 to 0.92) | Ref. | 0.67 (0.40 to 1.10) |
| Multivariate HR (95% CI)* | Ref. | 0.76 (0.58 to 0.98) | Ref. | 0.78 (0.47 to 1.29) |

*Adjusted for age, BMI (<25 kg/m^2^, 25-27.5 kg/m^2^, 27.5-30 kg/m^2^, ≥30 kg/m^2^) physical activity (<3 MET-hours/week, 3-27 MET-hours/week, ≥27 MET-hours/week), family history of colorectal cancer (no, yes), alcohol intake (<5 g/day, 5-15 g/day, ≥15 g/day), pack-years of smoking (0, 1-10, ≥10 pack-years), history of lower endoscopy (never/ever), caloric intake (quintiles), vitamin D (quintiles), calcium intake (quintiles), regular aspirin use (no, yes), folate intake (quintiles), MHT use (premenopausal, postmenopausal never user, postmenopausal past user, postmenopausal current user) (NHS and NHSII only), and red meat as main dish (quintiles)

Abbreviations: BMI, body mass index; CI, confidence interval; HR, hazards ratio; MHT, menopausal hormone therapy; NHS, Nurses’ Health Study; NHSII, Nurses’ Health Study II

**Supplementary Table 2**. Association between baseline use of proton pump inhibitors and H2-receptor antagonists with risk of colorectal cancer by selected patient characteristics

|  | **PPI use** | | | **H2RA use** | | |
| --- | --- | --- | --- | --- | --- | --- |
| **Characteristics** | Non-users | Users | *P*-interaction | Non-users | Users | *P*-interaction |
| **Gender** |  |  |  |  |  |  |
| *Women*  N  Person-years  HR (95% CI)* | 998  1,785,384  Ref. | 53  115,921  0.86 (0.65 to 1.14) |  | 1,486  16,888,427  Ref. | 56  917,684  0.70 (0.53 to 0.92) |  |
| *Men*  N  Person-years  HR (95% CI)* | 174  2,155,225  Ref. | 30  393,731  1.02 (0.68 to 1.52) | 0.63 | 837  705,765  Ref. | 22  17,711  1.01 (0.65 to 1.55) | 0.24 |
| **History of lower endoscopy** |  |  |  |  |  |  |
| *No*  N  Person-years  HR (95% CI)* | 512  772,852  Ref. | 21  32,908  0.84 (0.53 to 1.31) |  | 1,231  1,849,026  Ref. | 28  66,702  0.72 (0.49 to 1.05) |  |
| *Yes*  N  Person-years  HR (95% CI)* | 660  1,184,815  Ref. | 62  114,219  0.91 (0.70 to 1.19) | 0.85 | 1,092  1,738,613  Ref. | 50  104,022  0.77 (0.57 to 1.02) | 0.68 |
| **BMI** |  |  |  |  |  |  |
| *<25 kg/m^2^*  N  Person-years  HR (95% CI)* | 557  1,050,792  Ref. | 26  54,665  0.81 (0.54 to 1.20) |  | 1,058  1,941,225  Ref. | 29  66,832  0.79 (0.55 to 1.15) |  |
| *25-30 kg/m^2^*  N  Person-years  HR (95% CI)* | 411  595,549  Ref. | 36  55,335  0.97 (0.68 to 1.38) |  | 896  1,127,532  Ref. | 32  58,442  0.79 (0.55 to 1.13) |  |
| *≥30 kg/m^2^*  N  Person-years  HR (95% CI)* | 204  311,326  Ref. | 21  37,128  0.96 (0.60 to 1.53) | 0.48 | 369  518,882  Ref. | 17  45,450  0.75 (0.45 to 1.23) | 0.66 |
|  |  |  |  |  |  |  |
| **History of smoking** |  |  |  |  |  |  |
| *Never*  N  Person-years  HR (95% CI)* | 534  1,125,537  Ref. | 36  77,041  0.90 (0.64 to 0.27) |  | 1,046  2,044,206  Ref. | 31  87,443  0.75 (0.52 to 1.08) |  |
| *Ever*  N  Person-years  HR (95% CI)* | 638  832,130  Ref. | 47  70,086  0.90 (0.66 to 1.22) | 0.90 | 1,277  1,543,433  Ref. | 47  83,281  0.79 (0.59 to 1.06) | 0.93 |

*Adjusted for age, BMI (<25 kg/m^2^, 25-27.5 kg/m^2^, 27.5-30 kg/m^2^, ≥30 kg/m^2^) physical activity (<3 MET-hours/week, 3-27 MET-hours/week, ≥27 MET-hours/week) , family history of colorectal cancer (no, yes), alcohol intake (<5 g/day, 5-15 g/day, ≥15 g/day), pack-years of smoking (0, 1-10, ≥10 pack-years), history of lower endoscopy (never/ever), caloric intake (quintiles), vitamin D (quintiles), calcium intake (quintiles), regular aspirin use (no, yes), folate intake (quintiles), MHT use (premenopausal, postmenopausal never user, postmenopausal past user, postmenopausal current user) (NHS and NHSII only), and red meat as main dish (quintiles), excluding the stratifying variable

Abbreviations: BMI, body mass index; CI, confidence interval; H2-RA, H2-receptor antagonist; HR, hazards ratio; MHT, menopausal hormone therapy; NHS, Nurses’ Health Study; NHSII, Nurses’ Health Study II; PPI, proton pump inhibitor
